# Unraveling the Role of Red:Blue LED Lights on Resource Use Efficiency and Nutritional Properties of Indoor Grown Sweet Basil

**DOI:** 10.3389/fpls.2019.00305

**Published:** 2019-03-13

**Authors:** Giuseppina Pennisi, Sonia Blasioli, Antonio Cellini, Lorenzo Maia, Andrea Crepaldi, Ilaria Braschi, Francesco Spinelli, Silvana Nicola, Juan A. Fernandez, Cecilia Stanghellini, Leo F. M. Marcelis, Francesco Orsini, Giorgio Gianquinto

**Affiliations:** ^1^DISTAL – Department of Agricultural and Food Sciences and Technologies, Alma Mater Studiorum – Università di Bologna, Bologna, Italy; ^2^DISAFA-VEGMAP, Department of Agricultural, Forest and Food Sciences, University of Turin, Turin, Italy; ^3^Departamento de Producción Vegetal, Escuela Técnica Superior de Ingeniería Agronómica, Universidad Politécnica de Cartagena, Cartagena, Spain; ^4^Flytech s.r.l., Belluno, Italy; ^5^Wageningen UR Greenhouse Horticulture, Wageningen, Netherlands; ^6^Horticulture and Product Physiology Group, Wageningen University & Research, Wageningen, Netherlands

**Keywords:** *Ocimum basilicum* L., plant factories with artificial lighting (PFALs), water use efficiency (WUE), energy use efficiency (EUE), land surface use efficiency (SUE), nutrient use efficiency (NUE)

## Abstract

Indoor plant cultivation can result in significantly improved resource use efficiency (surface, water, and nutrients) as compared to traditional growing systems, but illumination costs are still high. LEDs (light emitting diodes) are gaining attention for indoor cultivation because of their ability to provide light of different spectra. In the light spectrum, red and blue regions are often considered the major plants’ energy sources for photosynthetic CO_2_ assimilation. This study aims at identifying the role played by red:blue (R:B) ratio on the resource use efficiency of indoor basil cultivation, linking the physiological response to light to changes in yield and nutritional properties. Basil plants were cultivated in growth chambers under five LED light regimens characterized by different R:B ratios ranging from 0.5 to 4 (respectively, RB_0.5_, RB_1_, RB_2_, RB_3_, and RB_4_), using fluorescent lamps as control (CK_1_). A photosynthetic photon flux density of 215 μmol m^−2^ s^−1^ was provided for 16 h per day. The greatest biomass production was associated with LED lighting as compared with fluorescent lamp. Despite a reduction in both stomatal conductance and PSII quantum efficiency, adoption of RB_3_ resulted in higher yield and chlorophyll content, leading to improved use efficiency for water and energy. Antioxidant activity followed a spectral-response function, with optimum associated with RB_3_. A low RB ratio (0.5) reduced the relative content of several volatiles, as compared to CK_1_ and RB ≥ 2. Moreover, mineral leaf concentration (g g^−1^ DW) and total content in plant (g plant^−1^) were influences by light quality, resulting in greater N, P, K, Ca, Mg, and Fe accumulation in plants cultivated with RB_3_. Contrarily, nutrient use efficiency was increased in RB ≤ 1. From this study it can be concluded that a RB ratio of 3 provides optimal growing conditions for indoor cultivation of basil, fostering improved performances in terms of growth, physiological and metabolic functions, and resources use efficiency.

## Introduction

Previsions on the increase of the global population (Wei and Ewing, 2018) suggest that agricultural land availability per capita (0.7 ha today) will decrease in the coming years (Chen et al., 2018). Agriculture nowadays uses about 70% of the world freshwater (Johnson et al., 2001) but both climate change and increase in human water use will likely result in water shortages (Elliott et al., 2014). Furthermore, the overall sustainability of current food systems is also constrained by the overuse of mineral fertilizers (Lal, 2018). As a consequence, the challenge agriculture has to face in the upcoming 50 years will be an increasing demand for food to feed ever larger cities with ever fewer resources.

In this scenario, new forms of agriculture that are not dependent on arable land and that can be developed also in the urban environment are gaining increasing popularity (Kalantari et al., 2017). Indoor farms, also called Plant Factories with Artificial Lighting (PFALs) or Vertical Farms with Artificial Lighting (VFALs), are closed plant production systems where environmental factors (e.g., temperature, humidity, light, and CO_2_ concentration) are controlled, minimizing the interactions with the external climate.

From a resource use perspective, VFALs present improved use efficiency of land, water, and nutrients (Graamans et al., 2018). By developing crop production in the vertical dimension, these systems can grow a higher number of plants per unit of cultivation area leading to a greater yield as compared with traditional cultivation systems (Kalantari et al., 2017). In VFALs, the adoption of hydroponics for growing plants and the possibility to recover water loss for transpiration results in up to 97% of water saving as compared to conventional agriculture (Kozai and Niu, 2016). Nutrient solution drained from the cultivation system can be recirculated avoiding run-off and leaching in soil (Dou et al., 2018). Finally, growing plants in an indoor environment enables to limit the entrance of pathogens or pests, which may allow for a pesticide-free production (Kalantari et al., 2017). To date, however, the large energy consumption associated with illumination, cooling, heating, and dehumidification, is limiting the diffusion of VFALs (Graamans et al., 2018).

From a physiological perspective, the effect of light on plant photosynthesis and growth is substantial. Specific portions of the spectrum are mostly important because the quantum yield curve presents two peaks at red and blue ranges (Inada, 1976) suggesting that they are the major energy sources for photosynthetic CO_2_ assimilation (Lin et al., 2013). The application of red and blue LEDs for indoor sweet basil cultivation was investigated by several researches in order to study how light quality can affect growth, photosynthetic activity, antioxidant capacity (Piovene et al., 2015; Bantis et al., 2016) and volatile profile (Carvalho et al., 2016). Nevertheless, the appropriate balance between red and blue components in the light spectrum for indoor cultivation of leafy vegetables and herbs remains unclear. The aim of this study is to identify how basil plants respond to variations in the red:blue (R:B) ratio of the incident light, providing a better understanding of physiological and biochemical adaptations set in order by the plants. The objective of the research is to foster a clearer comprehension of how functional physiological adaptations (including stomatal response and photosynthetic quantum yield efficiency) to light may be linked with changes in the plant biochemical profile (specifically for content of chlorophyll, minerals, antioxidants, and volatiles), overall leading to the definition of optimal light composition for increasing yield and resource use efficiency (e.g., water, energy, land surface, and mineral nutrition) in indoor basil cultivation.

## Materials and Methods

### Plant Material and Growth Conditions

Five separate experiments were conducted in growth chambers at the Universities of Bologna (Italy) and Wageningen (Netherlands). In all experiments, basil plants belonging to the typology “Genovese” (*Ocimum basilicum* cv. Superbo, Sais seeds, Cesena, Italy) were used, with a planting density of 100 plants m^−2^ (Saha et al., 2016). During all experiments, plants were grown under artificial light only, with measured photosynthetic photon flux density (PPFD) within the growth chambers of 215 ± 5.5 μmol m^−2^ s^−1^, a photoperiod of 16/8 h of light/dark and air temperature of 24 ± 2°C, with 55–70% of relative humidity and 450 ppm CO_2_. Details on the growing systems, irrigation and fertilization management are provided in the experiment description below.

#### Light Treatments

In all experiments, LED lamps (Flytech s.r.l., Belluno, Italy) featuring red (peak at 669 nm, OSRAM Hyper Red) and blue (peak at 465 nm, OSRAM Blue) diodes (OSRAM, 2017a,b) were used. LED treatments (spectra displayed in Supplementary Figure S1) were characterized by five R:B ratios, i.e., 0.5, 1, 2, 3, and 4 (respectively, indicated by RB_0.5_, RB_1_, RB_2_, RB_3_, and RB_4_). Specific features of all lamps are included in Table 1. Ratio between red and blue portions was calculated by defining the relative areas of the spectrum within the red (600–700 nm) and the blue (400–500 nm) regions (Singh et al., 2015). At the beginning of each experiment, lamps features were simultaneously measured as follows. Electricity absorbance (*W* = J s^−1^) was measured using a multimeter (Fluke 189, Fluke Corporation, Everett, WA, United States). Photosynthetic Photon Flux Density (PPFD, μmol m^−2^ s^−1^) was measured using a PAR Photon Flux Sensor model QSO (Apogee instruments, Logan, UT, United States) connected with a ProCheck handheld reader, manufactured by Decagon Devices Inc. (Pullman, WA, United States). According to the instruction manual, the PAR meter has similar sensitivity to both red and blue light. Photosynthetic Photon Number Efficacy (PPNE, μmol J^−1^), an indicator of the efficacy of converting electricity into light by the lamps (i.e., the output of photons per input of electricity), was calculated as the ratio between incident PPFD and the lamp electric absorbance (Akiyama and Kozai, 2016). The junction temperature of the diode (*T*_j_) is strongly linked with the relative luminous flux emitted by the diode (Poppe, 2015). *T*_j_ was calculated as follows:

**Table 1:** Main features of the lamps used in the experiments.

LAMP	Model, supplier	PPFD	Spectrum			Energy used	PPNE	*T*_j_-Blue	*T*_j_-red
		(μmol m^−2^ s^−1^)	Red (%)	Blue (%)	R:B ratio	(W m^−2^)	μmol J^−1^	°C	°C
RB_0.5_	Flygrow_0.5_, Flytech	215	30	58	0.5	154	1.40	61	50
RB_1_	Flygrow_1_, Flytech	215	46	44	1	172	1.25	62	53
RB_2_	Flygrow_2_, Flytech	215	62	30	2	210	1.02	65	65
RB_3_	Flygrow_3_, Flytech	215	70	23	3	219	0.98	65	65
RB_4_	Flygrow_4_, Flytech	215	75	19	4	219	0.98	65	70
CK_1_	TL-D90 De Luxe 950, Philips	215	29	31	1	386	0.55	–	–

*Photosynthetic Photon Flux Density (PPFD), spectral properties, energy used, photosynthetic photon number efficacy (PPNE), expressed as the ratio between the PPFD supplied (μmol m^−2^ s^−1^) and the electric energy consumed (J m^−2^), and calculated junction temperatures (T_j_) of the diodes are reported.*

$$T_{\text{j}}=T_{\text{c}}+{\mathrm{ThR}}_{\text{js}}*I_{\text{f}}*V_{\text{f}}$$

where *T*_c_ is the measured temperature on the pad (*T*_c_), ThR_js_ is the thermal resistance of the LED between the junction and the solder point (feature of the LED, being 9.8 K W^−1^ for blue and 5.3 K W^−1^, OSRAM, personal communication), *I*_f_ is the current on the LED, and *V*_f_ is the direct tension on the LED. Temperature of the pad was measured using an AMPROBE 38-XRA thermometer (Amprobe, NY, United States).

#### Experiments at Bologna University

Four experiments were consecutively conducted in six separate compartments (each 0.64 m^2^ surface and 0.4 m^3^ volume) of a climate controlled growth chamber at the Department of Agricultural and Food Sciences (DISTAL) of Bologna University, Italy. Each compartment was sealed with light opaque walls, white painted in the internal part, and equipped with fans constantly replacing internal air (hourly exchange rate of 200 v:v). Before each experiment, full randomisation of light treatments was operated.

Seeds were germinated in polystyrene containers filled with a mixture of peat (70%) and vermiculite (30%), under fluorescent lamps (CK_1_, see specifics in Table 1). When plants reached the two true leaf stage (3 weeks after sowing), roots were gently washed and plantlets were transplanted into individual hydroponic systems. Each single-plant hydroponic unit was composed of an hermetically sealed plastic jar (1 L of volume), filled with a nutrient solution (EC = 1.6, pH = 6.5) with the following composition: N-NO_3_: 14 mM; N-NH_4_: 4.4 mM; P: 1.0 mM; K: 5.0 mM; S: 2.0 mM; Ca: 1.2 mM; Mg: 5.2 mM; Fe: 17.9 μM, Cu: 2.0 μM, Zn: 3.8 μM, B: 11.6 μM, Mn: 18.2 μM, Mo: 0.5 μM. The nutrient solution was constantly aerated using air pumps (Airline 3, Haquoss, Turin, Italy, air exchange rate of 0.25 L min^−1^ pot^−1^) and was not substituted nor replenished before harvest.

Upon transplanting, six light treatments were applied, one per each compartment. In one compartment fluorescent lamps (CK_1_, TL-D 90 De Luxe 58W, Philips, Eindhoven, Netherlands) were installed, whereas the other five chambers were equipped with lamps with variable R:B ratio in the spectrum from 0.5 to 4 (using the lamps described in section “Light Treatments,” namely RB_0.5_, RB_1_, RB_2_, RB_3_, and RB_4_). Each compartment hosted 48 plants and measurements were taken in the central 9 plants. Each experiment was closed when commercial harvest was reached, at 18 Days After light Treatment (DAT), which meant 39 Days After Sowing (DAS).

#### Experiment at Wageningen University

One additional experiment was specifically designed to assess the PSII quantum efficiency (*F*_q_′/*F*_m_′) in response to the variable rates of red and blue fractions in the spectrum. This was performed at Wageningen University (Netherlands). The experiment took place in five separated compartments (each 0.4 m^3^) inside a climate controlled growth chamber. For the entire growth cycle a standard nutrient solution (EC = 2.0, pH = 6.0) was used to water the plants, using the following composition: N-NO_3_: 12.4 mM; N-NH_4_: 1.2 mM; P: 1.1 mM; K: 7.2 mM; S: 3.3 mM; Ca: 4.1 mM; Mg: 1.8 mM; Fe: 25 μM; Cu: 0.8 μM, Zn: 5 μM, B: 30 μM, Mn: 10 μM, Mo: 0.5 μM. The nutrient solution was supplied as ebb- and flow irrigation once a day in order to keep the plants well-watered at any time. Seeds were germinated in 6 cm pots filled with peat, under LED lamps (Greenpower led production module Deep Red/White 120, Philips, 28 W). When plants reached a two true leaf stage (21 DAS), five LED light treatments were used, resembling the same conditions used in the previous experiments, varying the R:B ratio in the spectrum from 0.5 to 4 (namely RB_0.5_, RB_1_, RB_2_, RB_3_, and RB_4_, using the same lamps manufactured by Flytech s.r.l., Belluno, Italy). Measurements of fresh yield, PSII quantum efficiency and chlorophyll were taken after 10 days of plants acclimation to the different light treatments (10 DAT), at 31 DAS.

### Vegetative and Physiological Measurements

#### Growth Analysis and Resource Use Efficiency

At harvest time, edible fresh weight (FW) of leaves and stem was measured in all experiments. Water use was individually quantified for each plant during experiments 1, 2, 3, and 4 and Water Use Efficiency (WUE) was calculated by the ratio between leaf fresh yield and the volume of water used, and expressed as g FW L^−1^ H_2_O. Lighting Energy Use Efficiency (EUE) was determined according to the crop cycle length and the final leaf yield, related to the lamps’ cumulated electricity absorption and expressed as g FW kW^−1^. Land Surface Use Efficiency (SUE) was determined by analyzing the potential achievable yield per unit land surface (1 m^2^, with plant density of 100 plants m^−2^) over a year (considering immediate transplanting of new plants after the harvest, resulting in 20 crop cycles each year). As for the hereby described experiments, recommended height of a layer may vary between 0.4 (Kozai, 2013; Kozai and Niu, 2016) and 0.5 m (Kozai, 2016). Three scenarios for SUE are therefore proposed, one on a single layer and two that consider vertical cultivation over multiple layers, representing, respectively, plant factories over 5 layers (Graamans et al., 2018) or over 10 layers (Kozai and Niu, 2016). Finally, crop Nutrient Use Efficiency (NUE) was calculated by the ratio between fresh weight and the total concentration of selected nutrients (N, P, K, Ca, Mg, and Fe) (Benincasa et al., 2011), analyzed in leaf tissues as described below.

#### Stomatal Conductance

Measurements of stomatal conductance (μmol m^−2^ s^−1^) were performed on the third fully expanded leaf from the apex using a leaf porometer (AP4, Delta-T Devices, Cambridge, United Kingdom) at 18 and 11 DAT on experiments 3 and 4, respectively.

#### PSII Quantum Efficiency

Measure of PSII quantum efficiency (*F*_q_′/*F*_m_′) was performed in experiment 5, using a PlantExplorer^TM^ (PhenoVation B.V., Wageningen, Netherlands), at 10 DAT (31 DAS). This parameter indicates the efficiency at which light absorbed by PSII is used for the primary quinone acceptor (*Q*_A_) reduction. At a given photosynthetically active photon flux density, this parameter provides a rapid method to determine the PSII operating efficiency under different light spectra (Baker, 2008). The instrument was equipped with the same lamps used during the experiments (namely RB_0.5_, RB_1_, RB_2_, RB_3_, and RB_4_, Flytech s.r.l., Belluno, Italy), which were kept on before and during measurements. Measurements were performed during 1 s, imposing an intensive light flash of 3500 μmol m^−2^ s^−1^ by a monochromatic red LED source (peak 660 nm).

#### Leaf Chlorophyll Content

Leaf chlorophyll content was estimated during experiments 1, 2, 3, and 4 at 10 DAT using a hand-held leaf chlorophyll meter (YARA N-Tester, Oslo, Norway) on the third fully expanded leaf from the apex. *N*-Tester calculates a numeric three-digit dimensionless value that is commonly expressed as *N*-Tester value (Orsini et al., 2018).

#### Whole Plant Chlorophyll Content (Chlorophyll Index)

Estimation of chlorophyll concentration was determined during experiment 5 through estimation of greenness using the bidirectional reflectance distribution function (BRDF) (Comar et al., 2012; Virlet et al., 2017), and obtained using PlantExplorer^TM^ (PhenoVation B.V., Wageningen, Netherlands) at 10 DAT (31 DAS). The measures were taken while imposing a white light from a LED lamp (2700 Kelvin, 150 μmol m^−2^ s^−1^).

### Biochemical Determinations

During experiments 2 and 3, basil leaf samples were immersed in liquid nitrogen and kept at −80°C for biochemical analysis, whereas other samples were dried at 58°C for 72 h in a ventilated oven for elemental analysis. Dry weights were recorded at this stage. Volatile compounds analysis were performed on fresh basil leaf samples during experiment 4.

#### Phenolic Compound Extraction

One gram of frozen samples of basil leaves was ground with liquid nitrogen and homogenized with 4 mL of methanol/H_2_O/acetone (60:30:10 v/v/v) (Hartmann et al., 2008). The mixture was centrifuged (Beckman Coulter, BECKMAN, El Cajon, CA, United States) at 15,300 × *g* for 10 min at 4°C and the supernatant was collected. The extraction was repeated twice and the final extract was used for the determination of total phenolic and flavonoid content and total antioxidant capacity.

#### Determination of Total Phenolic and Flavonoid Contents

Total phenolic content (TPC) was determined according to the Folin–Ciocalteu colorimetric method (Waterhouse, 2002). The total phenolic content was expressed as gallic acid equivalent in milligram per gram of fresh weight of leaves.

Total flavonoid content (TFC) was determined by aluminum chloride colorimetric assay (Zhishen et al., 1999). The results were expressed as mg of catechin equivalents per grams of fresh weight of leaves.

#### Total Antioxidant Capacity

Total antioxidant capacity was measured by Ferric Reducing Antioxidant Power (FRAP) assay, following methodologies from Benzie and Strain (1999) and Aaby et al. (2007). FRAP values were expressed as mmol Fe^2+^ kg^−1^ FW.

#### Nutrient Content Analysis

To assess a nutrient balance, micro- and macro-nutrient concentrations were measured both in nutrient solutions and in basil leaves. Leaf samples to be analyzed for nutrient content were collected at harvesting during experiments 2 and 3. Nutrient solutions were also collected at both transplanting stage and at the harvest. Micro- and macronutrients analyses of nutrient solutions were performed by using an inductively coupled plasma optical emission spectrometer ICP-OES equipped with a plasma source and an optical detector with a charge-coupled device CCD (SPECTRO Analytical Instruments GmbH & Co., Kleve, Germany), following the methodology presented in Lavrnić et al. (2018). Minerals uptake from nutrient solution was calculated by subtracting the concentration in the nutrient solution at the end of the cycle from the concentration in the fresh nutrient solution.

#### Total Nitrogen (TN) Analysis

Nutrient solutions (experiments 2 and 3) were analyzed for total nitrogen (TN) by using the elemental analyser Shimadzu TNM-1 (Shimadzu, Kioto, Japan). Before analysis, samples were filtered through Whatman 42 filters. TN content in dry leaf samples was measured by using a thermo-electron CHNS-O elemental analyser (Thermo Fisher Scientific, Waltham, MA, United States). The analysis was performed on ball-milled samples, following the methodology presented in Lavrnić et al. (2018).

#### Volatile Organic Compound (VOCs) Analysis of Basil Leaves

Volatile profile of basil leaves was determined in experiment 3 by gas chromatography coupled with mass spectrometry (GC–MS, QP-2010 Plus, Shimadzu, Japan), interfaced with a computerized system for data acquisition (Software GC–MS Solution V. 2.5, Shimadzu, Japan), following methodology presented in Cardenia et al. (2015). The identification of VOCs was achieved by comparing their mass spectra with those stored in the National Institute of Standards and Technology (NIST08) United States Government library and those reported in the literature. For each light treatment, GC-MS analysis was performed in triplicate using new fresh leaves as sample. Relative amounts of detected VOCs emitted by fresh basil leaves were calculated by integration of peak area followed by normalization to sample fresh weight. Data were expressed as mean of three replicates and standard deviations were calculated.

### Statistical Analysis

Measures were conducted on nine plants per light treatments, which were surrounded by border plants. For the measured parameters from experiments 1, 2, 3, and 4, data were analyzed by two-way ANOVA (light spectrum × experiment) and the means were compared by Least Significance Difference (LSD), at 5% significance level (Supplementary Table S1). Results from experiment 5 (fresh yield, chlorophyll index and PSII quantum efficiency) were analyzed using one-way ANOVA. Volatile compounds, measured only during experiment 3, were analyzed as the Log_2_ of the relative fold change from control treatment (CK_1_).

## Results

### Plant Growth and Input Use Efficiency

**FIGURE 1:**
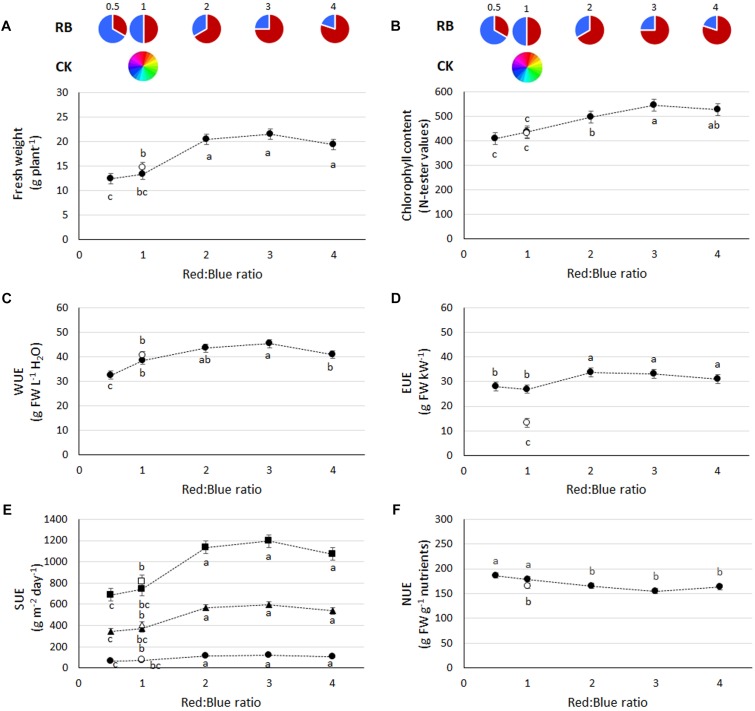
Growth and resources use efficiency in basil plants grown under LED lights with different R:B ratio (closed symbols) or under fluorescent lights (open symbols). **(A)** Fresh weight, **(B)** chlorophyll content, **(C)** water use efficiency (WUE), **(D)** Energy Use Efficiency (EUE), **(E)**, land Surface Use Efficiency (SUE) of a single layer (circles), or a vertical farming structure of five-layers (triangles) and ten-layers (square), and **(F)** overall Nutrient Use Efficiency (NUE) (based on total content of N, P, K, Ca, Mg, and Fe and expressed as g FW g^−1^ nutrients). Data referring to experiments 1, 2, 3, and 4 (charts **A–E**, 36 replicate plants) and experiments 2 and 3 (chart F, 18 replicate plants) are indicated as mean ± SE. Different letters indicate significant differences at *P* ≤ 0.05.

**FIGURE 2:**
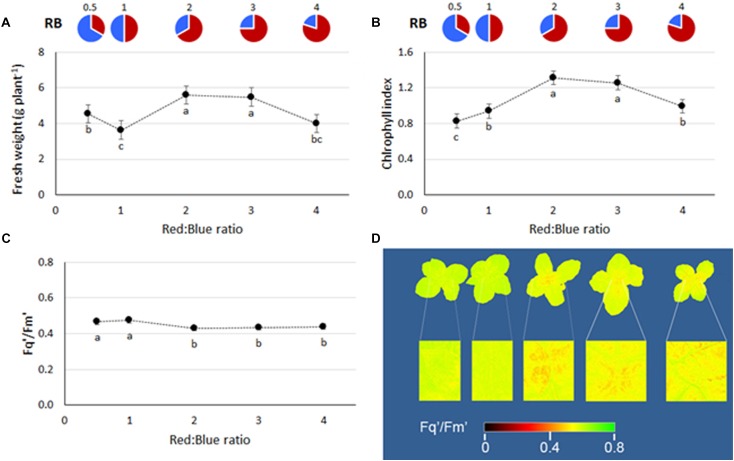
**(A)** Fresh weight, **(B)** chlorophyll index, and **(C)** PSII quantum efficiency of basil plants grown under LED lights with different R:B ratio in the spectrum (RB_0.5_, RB_1_, RB_2_, RB_3_, and RB_4_). The data, referring to experiment 5, are presented as mean values ± SE (15 replicate plants). Different letters indicate significant differences at *P* ≤ 0.05. In **(D)**, whole plant images created using PlantExplorer^TM^ are included. The greater presence of red pixels indicates lower values of *F*_q_′/*F*_m_′, whereas higher yellow/green areas indicate higher *F*_q_′/*F*_m_′ values, as defined in the integrated legend.

During the first four experiments (those conducted at Bologna University), no significant interactions between light and experiment were observed (ANOVA results in Supplementary Table S1), therefore average values from the four experiments are used for data representation in figures and tables. Plant fresh weight was enhanced with increasing RB ratio, reaching the highest values at RB ≥ 2 (Figure 1A). A consistent trend in plant chlorophyll content was also evident, with the highest values found when plants were grown at RB ≥ 3 (Figure 1B). Similar performances in terms of both yield and chlorophyll content in response to light were also evident during experiment 5 (conducted at Wageningen University), although a decrease in both yield and chlorophyll was observed at RB_4_ (Figure 2A,B).

From a resource use perspective, water used per plant varied from 0.34 (RB_1_ and CK_1_) to 0.42 (RB_0.5_ and RB_4_) and 0.48 (RB_2_ and RB_3_) L plant^−1^ (data not shown). Light energy used was highest in CK_1_ as compared with LED treatments (Table 1). The photosynthetic photon number efficacy (PPNE) values were lower in CK_1_ as compared with LEDs. Moreover, the lower the fraction of red light of the LEDs the higher the efficacy (Table 1). The *T*_j_ values were of 61–65°C for the blue LED and 50–70°C for the red LED (Table 1). Accordingly, among LEDs, an increase in electricity consumption was associated with the increase in the red portion of the spectrum from 154 to 219 W m^−2^ (respectively, in RB_0.5_ and RB_3_) (Table 1). However, with consideration to the plant capacity to transform resources, water use efficiency (Figure 1C) was the greatest in plants grown under RB ratio of 2 or 3 (average value of 44.5 ± 1.2 g FW L^−1^ H_2_O) and energy use efficiency (Figure 1D) was highest when RB ≥ 2 (average value of 32.7 ± 1.0 g FW kW^−1^).

As evidenced by the crop yield, SUE was increased with RB ≥ 2. When the number of layers was increased to 5 and 10 vertical tiers, achievable basil yield under RB ≥ 2 were, respectively, above 550 and 1100 g m^−2^ day^−1^ (Figure 1E).

The greatest nutrients removal from the nutrient solution was obtained by basil plants grown under LED lighting as compared to fluorescent light, except for the calcium uptake (non-affected by light regime) and the iron uptake, that was higher in both RB_4_ and CK_1_. In the leaf tissue, concentrations presented higher values in plants grown under RB_3_ as compared with CK, with exception of Mg that was most concentrated in leaf tissues of CK_1_ grown plants (Table 2) and K, which presented similar values in RB ≥ 3 and CK_1_. No difference in N concentration was associated with R:B ratio. Based on the overall mineral content per plant, N, P, K, Ca, Mg, and Fe were the highest in RB_3_, with comparable values observed in plants grown under RB_2_ (N, P, K, Ca, and Mg) or RB_4_ (N, K, and Mg). From a resource use perspective, NUE was the lowest when CK_1_ or LED lights with RB ≥ 2 were used, and this was mainly associated with higher concentrations in leaf tissue of the elements (Figure 1F).

**Table 2:** Nutrient solution uptake, leaf concentration and plant content of selected mineral elements in basil grown under fluorescent light (CK_1_) or LED lights at varying R:B ratio (RB_0.5_, RB_1_, RB_2_, RB_3_, and RB_4_).

	N						P						K					
	Uptake %		Concentration (mg g^−1^ FW)		Content (mg plant^−1^)		Uptake %		Concentration (mg g^−1^ FW)		Content (mg plant^−1^)		Uptake %		Concentration (mg g^−1^ FW)		Content (mg plant^−1^)	
RB_0.5_	36	c	**5.34**	**ab**	65.6	bc	**59**	**ab**	**1.09**	**ab**	13.4	bc	55	c	2.43	c	29.9	bc
RB_1_	34	c	**5.62**	**ab**	86.5	b	55	b	0.96	bc	14.9	b	52	c	2.47	c	38.0	b
RB_2_	41	bc	**5.67**	**ab**	**121.2**	**ab**	56	b	**1.13**	**ab**	**24.4**	**ab**	64	b	2.72	b	**58.9**	**a**
RB_3_	**52**	**a**	**5.83**	**a**	**143.6**	**a**	**64**	**a**	**1.19**	**a**	**29.3**	**a**	61	b	**2.89**	**a**	**71.8**	**a**
RB_4_	44	b	**5.80**	**a**	**111.9**	**ab**	57	b	1.05	b	19.6	b	**75**	**a**	**2.79**	**ab**	**53.1**	**ab**
CK_1_	27	d	5.10	b	36.0	c	48	c	0.89	c	6.7	c	41	d	**2.85**	**a**	21.9	c
	^∗∗∗^		^∗^		^∗∗∗^		^∗∗∗^		^∗∗∗^		^∗∗∗^		^∗∗∗^		^∗∗∗^		^∗∗∗^	

	**Ca**						**Mg**						**Fe**					
	**Uptake %**		**Concentration (mg g^−1^ FW)**		**Content (mg plant^−1^)**		**Uptake %**		**Concentration (mg g^−1^ FW)**		**Content (mg plant^−1^)**		**Uptake %**		**Concentration (μg g^−1^ FW)**		**Content (μg plant^−1^)**	
RB_0.5_	57		0.73	c	8.9	c	26	b	0.57	c	7.0	b	71	c	9.88	c	127.8	c
RB_1_	52		0.92	b	14.6	bc	20	c	0.66	c	10.2	b	79	b	8.76	c	135.9	c
RB_2_	50		0.92	b	**20.0**	**ab**	22	bc	0.69	bc	**14.7**	**ab**	77	b	12.93	b	284.4	b
RB_3_	55		**1.06**	**a**	**26.3**	**a**	**29**	**ab**	0.73	bc	**18.4**	**a**	78	b	**17.32**	**a**	**439.4**	**a**
RB_4_	55		**0.94**	**ab**	18.8	b	**33**	**a**	0.79	b	**15.2**	**ab**	**91**	**a**	11.32	bc	230.2	bc
CK_1_	52		0.88	b	6.8	c	22	bc	**0.92**	**a**	7.1	b	**91**	**a**	7.76	c	59.0	c
	ns		^∗∗^		^∗∗∗^		^∗∗∗^		^∗∗∗^		^∗∗^		^∗∗∗^		^∗∗∗^		^∗∗∗^	

*Mean values from experiments 2 and 3 based on 18 replicate plants. Different letters indicate significant differences at ^∗^P ≤ 0.05, ^∗∗^P ≤ 0.01, ^∗∗∗^P ≤ 0.001. ns, not significant differences. Highest values are in bold.*

### PSII Quantum Efficiency (*F*_q_′/*F*_m_′)

The plant PSII quantum efficiency (measured during experiment 5) was lower with RB ≥ 2 whereas highest values were associated with RB ≤ 1 (Figure 2C). The reduction in *F*_q_′/*F*_m_′ was also visible from the acquired plant images, where a greater number of pixels presented lowered *F*_q_′/*F*_m_′ values (range of 0.32–0.48, in red), as presented in Figure 2D.

### Leaf Gas Exchanges

**FIGURE 3:**
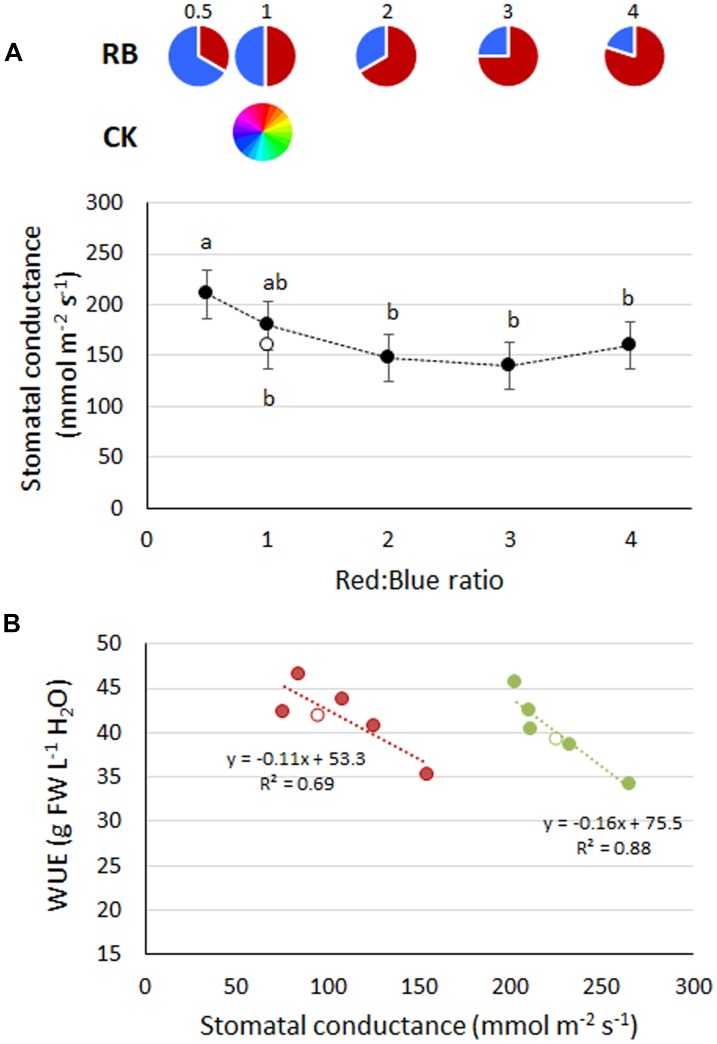
**(A)** Stomatal conductance in basil plants grown under LED lights with different R:B ratio in the spectrum or under fluorescent light (open symbol). Mean values (experiments 3 and 4) ± SE (18 replicate plants). Different letters indicate significant differences at *P* ≤ 0.05. **(B)** Linear relationship in experiment 3 (red) and experiment 4 (green) between mean values of stomatal conductance and Water Use Efficiency (WUE). Open symbols represent CK_1_.

The lowest stomatal conductance was observed in RB ≥ 2, with comparable values to CK_1_ and RB_1_ (Figure 3A). A greater presence of blue light in the spectrum (e.g., RB_0.5_) resulted in highest stomatal conductance (Figure 3A). A linear inverse relationship between stomatal conductance and water use efficiency was always observed (*R*^2^ of 0.69 and 0.88 in experiments 3 and 4, respectively) (Figure 3B).

### Antioxidant Properties

**FIGURE 4:**
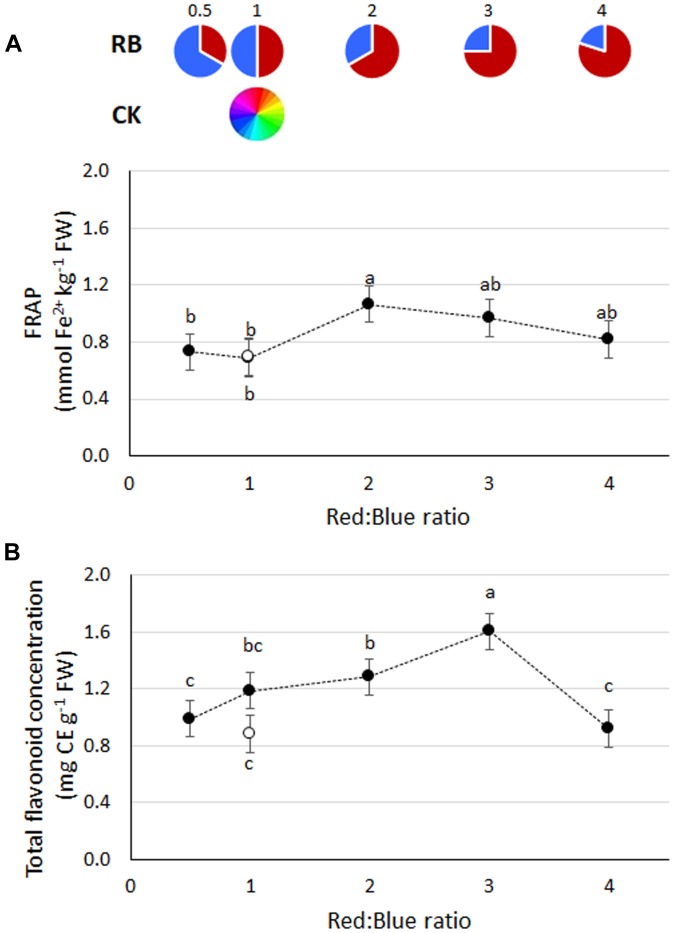
**(A)** Antioxidant capacity (FRAP) and **(B)** total flavonoids concentration in basil plants grown under LED lights with different R:B ratio in the spectrum or under fluorescent light (open symbol). Data referring to experiments 2 and 3 are indicated as mean ± SE (18 replicate plants). Different letters indicate significant differences at *P* ≤ 0.05.

Highest FRAP values were found in plants grown under RB_2_ (Figure 4A), although with comparable values to RB ≥ 3. Total flavonoid content was highest in RB_3_ (1.60 mg CE g^−1^ FW), followed by RB_1_ and RB_2_ (mean value of 1.23 mg CE g^−1^ FW) and by RB_0.5_, RB_4_, and CK_1_ (mean value 0.93 mg CE g^−1^ FW) (Figure 4B). No statistical differences in polyphenols content were observed in response to R:B ratio (data not shown).

### Aromatic Profile

**FIGURE 5:**
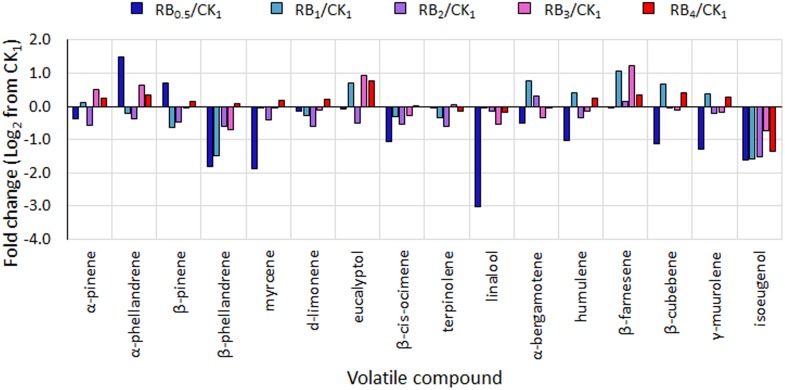
Fold change from control treatment (fluorescent light, CK_1_) in the amount of selected volatiles in basil leaves from plants grown under LED lights with different R:B ratio in the spectrum (RB_0.5_, RB_1_, RB_2_, RB_3_, and RB_4_). Mean values of 3 replicate plants per treatment (experiment 3) ± SE (3 replicate plants).

In basil leaves, 42 VOCs were identified (Supplementary Table S2), belonging to 7 chemical classes, namely alcohols, esters, hydrocarbons, monoterpenes, phenylpropanoids, sesquiterpenes, and terpenoids. Among these, only 16 VOCs were responsible for around 90% of variations in the aromatic profile (listed in Table 3). The predominant compounds found were linalool (in all treatments except RB_0.5_), and α-bergamotene (with greatest values observed in RB ≤ 2) (Table 3). When compared to values obtained under control light (CK_1_), RB_0.5_ caused a reduction in α-pinene, β-phellandrene, myrcene, β-*cis*-ocimene, linalool, α-bergamotene, humulene, β-cubebene, γ-murolene, and isoeugenol (Figure 5). On the other hand, RB ≥ 3 presented in most cases higher or similar relative values to those measured in CK_1_, with the only exception of isoeugenol, which was relatively lower in all LED treated plants.

## Discussion

### Obtaining Greater Yield and Better Nutritional Properties With the Appropriate R:B Balance in the Light Spectrum

Unraveling the optimal spectral recipe for indoor cultivation is the subject of a number of recent researches (Lin et al., 2013; Son and Oh, 2013; Piovene et al., 2015). In the present study, yield reduction was associated with an higher fraction of blue light, a phenomenon that was previously associated with lower internode length and smaller leaf area (Figure 1, 2) (Yanagi et al., 1996; Ménard et al., 2006; Chen et al., 2014). Contradictory results in these terms also exist, where the presence of blue light was reported not to alter basil plant height or fresh weight (Carvalho et al., 2016; Schwend et al., 2016). However, the replicability of research results in LED lighting applications is constrained by the great variability in the experimental setups (Massa et al., 2008; Morrow, 2008), with inconsistencies in existing literature associated with differences in the growing conditions, to be related with: (a) low PPFD values (e.g., 60–120 μmol m^−2^ s^−1^, Carvalho et al., 2016; Schwend et al., 2016), as compared with recommended values of 220 μmol m^−2^ s^−1^ for indoor basil production (Dou et al., 2018); (b) limited plant densities (e.g., down to 24 plants m^−2^, Piovene et al., 2015), as compared with common densities in hydroponics of 100 plants m^−2^ (Abbas, 2014); (c) changes in spectral composition (e.g., R:B ratio of 10 and inclusion of far-red radiation, Schwend et al., 2016); (d) long cycle length (e.g., up to 100 days, Schwend et al., 2016), against common cycles of about 40 days (Dou et al., 2018).

The yield increase (Figure 1A, 2A) and the higher leaf chlorophyll concentration (Figure 1B, 2B) associated with RB_3_, however, were not linked with increased photosynthetic efficiency (which was instead higher on RB ≤ 1, Figure 2C). In previous experiments, chlorophyll content was greater in LED illuminated grape leaves when red light instead of blue light was supplied (Wang et al., 2016). Similarly, a LED light supplying RB = 6 increased total chlorophyll in Chinese cabbage leaves as compared to the concentration observed in plants grown under blue or red light only (Fan et al., 2013). Moreover, in lettuce, leaf chlorophyll concentration was higher when plants were grown under a mixture of red and blue light (RB = 1), instead of blue or red light only (Chen et al., 2014). With regard to quantum efficiency of the PSII, experiments on cucumber (Hogewoning et al., 2010) suggested that variations in the R:B ratio have limited effects on *F*_q_′/*F*_m_′, although with significant decreases when the red portion was increased from 50% to 85, 93, and 100%. Furthermore, it was also shown that, similarly to the experimental evidences hereby described, increases in the red light fraction are associated with a concurrent decrease in stomatal conductance (Hogewoning et al., 2010), whose inverse relationship with WUE in basil (Figure 3B) was also previously described (Barbieri et al., 2012). Given that photosynthetic quantum yield efficiency could only be measured in one experiment (experiment 5), the observed trend needs to be substantiated by further investigations.

**Table 3:** Amount of main volatile compounds (mean percentage of total volatile content) in leaves of basil plants grown under fluorescent light (CK_1_) or LED lights at varying R:B ratio (RB_0.5_, RB_1_, RB_2_, RB_3_, and RB_4_).

Compound	RT (min)	CK_1_ (%)	RB_0.5_ (%)	RB_1_ (%)	RB_2_ (%)	RB_3_ (%)	RB_4_ (%)
α-pinene	4.026	0.5 ± 0.3	1.4 ± 0.8	0.5 ± 0.2	0.3 ± 0.0	0.9 ± 0.3	0.6 ± 0.2
α-phellandrene	4.158	0.3 ± 0.1	2.0 ± 0.9	0.2 ± 0.0	0.2 ± 0.1	0.5 ± 0.2	0.3 ± 0.1
β-pinene	6.360	0.8 ± 0.4	3.2 ± 1.2	0.4 ± 0.2	0.5 ± 0.1	0.9 ± 0.3	0.8 ± 0.3
β-phellandrene	6.993	2.2 ± 0.8	3.2 ± 2.0	0.8 ± 0.4	1.3 ± 0.2	2.1 ± 1.1	2.0 ± 0.2
Myrcene	9.573	3.6 ± 0.3	3.9 ± 2.4	3.0 ± 0.5	2.9 ± 0.3	4.7 ± 1.7	4.0 ± 0.4
Terpinolene	10.055	1.7 ± 0.4	3.4 ± 0.4	1.1 ± 0.2	1.1 ± 0.2	2.3 ± 0.8	1.5 ± 0.3
D-limonene	11.196	1.8 ± 0.4	3.4 ± 0.4	1.2 ± 0.2	1.2 ± 0.1	2.0 ± 0.5	1.9 ± 0.3
Eucalyptol	11.592	1.0 ± 0.4	2.2 ± 0.8	2.4 ± 0.1	0.7 ± 0.1	2.3 ± 1.0	1.7 ± 0.6
β-*cis*-ocimene	14.589	7.0 ± 3.9	12.9 ± 1.9	8.5 ± 1.8	9.1 ± 1.7	12.2 ± 2.4	11.9 ± 1.1
**Linalool**	**26.322**	**44.6 ± 1.7**	**12.8 ± 3.1**	**37.1 ± 3.4**	**42.7 ± 6.0**	**35.9 ± 2.6**	**38.6 ± 5.1**
humulene	29.576	0.9 ± 0.1	1.0 ± 0.3	1.0 ± 0.2	0.7 ± 0.1	1.0 ± 0.2	1.4 ± 0.8
β-farnesene	30.000	1.7 ± 0.3	3.8 ± 0.9	3.0 ± 0.5	2.4 ± 1.0	5.1 ± 1.3	2.5 ± 1.0
β-cubebene	30.770	2.4 ± 0.3	2.5 ± 0.6	3.3 ± 0.9	2.4 ± 0.1	2.6 ± 0.3	3.5 ± 1.3
**α-bergamotene**	**31.959**	**17.8 ± 1.1**	**28.6 ± 5.9**	**25.1 ± 1.6**	**23.5 ± 2.5**	**17.0 ± 3.4**	**16.9 ± 1.6**
γ-muurolene	32.272	3.2 ± 0.5	3.0 ± 0.8	3.6 ± 0.9	2.8 ± 0.2	3.2 ± 0.4	3.9 ± 1.1
Isoeugenol	42.402	2.1 ± 1.0	1.4 ± 0.7	0.4 ± 0.1	0.6 ± 0.2	1.2 ± 0.3	0.6 ± 0.2
Percentage of total included		91.6 ± 0.6	88.7 ± 2.0	91.6 ± 1.3	92.4 ± 2.1	93.9 ± 1.0	92.2 ± 1.8

*Mean values of 3 replicate plants per treatment (experiment 3) ± SE. In bold, most represented volatiles.*

Beside yield, qualitative parameters of indoor grown crops are of great relevance since they directly affect the product economic value (Kopsell and Sams, 2013; Lin et al., 2013). Light quality was reported to trigger the synthesis of secondary metabolites in herbs (Koseki et al., 2002), but so far no effects of red to blue ratio were observed in basil (Piovene et al., 2015; Carvalho et al., 2016). Similarly, no differences in FRAP, polyphenol and flavonoid content were observed in basil grown under red and blue lamps providing R:B ratios from 0.7 to 5.5 (Piovene et al., 2015). On the other hand, red light was also shown to improve antioxidant capacity in both lettuce and basil (Samuolienė et al., 2012, 2016). In the present study, the accumulation of functional compounds, e.g., antioxidants, was achieved by modifying the light spectrum, as evidenced in the case of RB_3_, where values of both FRAP and flavonoid content were the highest as compared with RB ≤ 1 (Figure 4). If this is combined with the observed yield increase associated with RB_2_ and RB_3_, the amount of functional compounds achievable per plant is by far greater than in the cases of RB_0.5_ or CK_1_. These evidences support the hypothesis that, when red light increases up to RB_3_, photoprotective mechanisms resulted in lower photosynthetic efficiency (Hogewoning et al., 2010) and greater antioxidant biosynthesis (Samuolienė et al., 2016).

With regard to the leaf aromatic profile, α-bergamotene (sesquiterpenes) and linalool (terpenoids) were found to be among the predominant VOCs, as for previous literature (Carvalho et al., 2016) (Table 3). The relative reduction in linalool (Figure 5) observed in RB_0.5_, as compared with all other treatments, could be associated with a delayed growth (Figure 1A, Carvalho et al., 2016) and the concurrent effect of abiotic stresses, such as unbalanced light supply (Loughrin and Kasperbauer, 2003; Carvalho et al., 2016). Since these measurements were only performed in experiment 3, however, these conclusions need further investigations in order to be confirmed.

### The Role of Spectral Composition in the Resource Use Efficiency of Indoor Plant Cultivation

#### Water Use Efficiency

The water saving potential associated with the adoption of protected cultivation and hydroponics is a well-established concept (Gruda, 2009; Putra and Yuliando, 2015). WUE values of traditional open field cultivation of basil were reported to be as low as 3 g FW L^−1^ H_2_O in Turkey (Ekren et al., 2012). Contrarily, WUE reached 20–22 g FW L^−1^ H_2_O when potted basil was grown in greenhouses in Italy (Montesano et al., 2018). In the present experiments, WUE increased up to above 45 g FW L^−1^ H_2_O in RB_2_ and RB_3_ (Figure 1C), thanks to a higher biomass production (Figure 1A) despite a slight increase in water use (data not shown). This value could possibly increase further in real VFALs, where potentially all transpired water may be recovered and re-circulated (Kozai, 2013). Beside the role of the cultivation system itself in water saving, differences were also evident among light treatments (Figure 1C). These, are to be associated with changes in the stomatal behavior (Figure 3). It is generally acknowledged that blue light (Kinoshita et al., 2001) is more effective than red light in promoting stomata opening (Shimazaki et al., 2007). Addition of blue light to background red light was reported to increase stomatal density in leaves of grape (Poudel et al., 2008), cherry tomato (XiaoYing et al., 2011), and also basil (Jensen et al., 2018). In the present study, in plants grown under RB ≥ 2, WUE was improved by +25% (Figure 1C). Accordingly, a more effective water use by the plant was achieved through a response to the higher red light fraction in the spectrum, which led to preserved plant growth beside the induced stomatal closure (Baroli et al., 2008).

#### Energy Use Efficiency

The electricity cost associated with lighting is one of the main components of the production costs in VFALs (Kozai, 2013). Beside the electricity rate (that varies in each country) and the lamps efficiency to convert electricity into light, the plant capability to convert the provided radiation into effective growth will affect the VFAL sustainability. In LED lights, the photosynthetic photon number efficacy (PPNE) was reported to be between 0.5 and 2.3 μmol J^−1^ (Nelson and Bugbee, 2014; Akiyama and Kozai, 2016; Park and Runkle, 2018). Alternatively, fluorescent lamps generally show a 35% lower PPNE as compared with LED (Yano, 2016). PPNE were reported to be higher in blue LED (1.9 μmol J^−1^) as compared with red LED (1.7 μmol J^−1^) (Nelson and Bugbee, 2014). Another study (Wallace and Both, 2016), compared PPNE in LED lamps from different manufacturers, presenting values of 1.4 μmol J^−1^ (Valoya R150 NS1, RB = 2, PPFD = 184 μmol m^−2^ s^−1^), 0.8 μmol J^−1^ (Orbitec LED tower, RB = 3, PPFD = 84 μmol m^−2^ s^−1^) and 1.4 μmol J^−1^ (Cree 18W Daylight, RB = 1.3, PPFD = 4 μmol m^−2^ s^−1^). PPNE was also shown to be around 1.3 μmol J^−1^ in a red-blue top-light (Valoya AP-67, RB = 3, PPFD = 160 μmol m^−2^ s^−1^) (Särkkä et al., 2017). Nájera et al. (2018) reported PPNE to range 0.9 (Valoya AP-67, RB = 3, PPFD = 64 μmol m^−2^ s^−1^) to 1.2 (Valoya AP673, RB = 5, PPFD = 85 μmol m^−2^ s^−1^), although details in the measurements performed for quantifying electricity consumption or R:B ratio were lacking. In the present experiments, PPNE increased when the fraction of blue increased. This trend may conflict with the common belief that red diodes are more efficient than blue ones in converting electricity into PAR (Schulze et al., 2014). Accordingly, red and blue LEDs (at 450 mA amperage), were stated to have efficiencies, respectively, of 2.3 and 1.8 μmol J^−1^ (Park and Runkle, 2018). Similar values were also observed in an older study (Blanken et al., 2013), with efficiencies of 2.6 (red) and 2.0 (blue) μmol J^−1^ (Philips Luxeon), but once again PPNE values were based on manufacturer datasheet rather than measured in the experimental environment. The scientific explanation behind the higher efficacy of red versus blue LED is that more photons are usually released by LEDs emitting at longer wavelengths (e.g., red), resulting in higher PPNE than for LEDs emitting at shorter wavelengths (e.g., blue) (Blanken et al., 2013), because blue photons are more energetic than red ones. However, as evidenced by Wang et al. (2007), the PPNE varies also in response to the use intensity of the light source and the consequential changes in its junction temperature. For instance, efficiency of red LED is reported to drop more at high temperature than that of blue, according to most LED manufacturers. Accordingly, when the temperature of LED increases from 25 to 60°C the light output of red and blue LEDs drops by 5–15% and 3–5%, respectively (LUMILED, 2016, 2017; CREE, 2017). Coming to the LED used in this study, the relative radiant power at 350 mA decreases as temperature increases from 25 to 50–55°C (−7%) and 65–70°C (−12%) in the “Hyper red” LED (peak 646–666 nm) (OSRAM, 2017b). Moreover, as temperature in the “Blue” LED (peak 464–476 nm) was raised from 25°C to 60–65°C, a concurrent increase in measure of +7% in the relative luminous flux was also observed (OSRAM, 2017a). The decreased PPNE upon RB ≥ 1 could therefore be associated with the observed *T*_j_ values that would have resulted in increased efficacy of blue vs. red diodes. Such variations tended to stabilize as the red portion of the spectrum increase, and PPNE was previously assumed not to vary (e.g., PPNE = 2.15 μmol J^−1^) when the R:B ratio was varied (from RB = 1 to RB = 2.3, PPFD = 62.5 μmol m^−2^ s^−1^) (Hernández et al., 2016) as evidenced in the present study in RB ≥ 1. However, based on the crop growth, it should be noted that the EUE was greater when higher spectral portions were allocated to the red region as for the case of RB ≥ 2 (Figure 1D), due to a larger yield increase observed in these treatments compared to RB ≤ 1.

#### Land Surface Use Efficiency

Increased SUE in VFALs is claimed to be from 10- to 100-folds (Kozai, 2013) higher as compared to greenhouses or open field, due to increased plant growth rates, vertical distribution of the growing system and high yields. Preliminary estimates based on application of combined mathematical models on lettuce productivity per unit cropped area, suggest that in VFALs yield would be 2.5 higher than in a greenhouse in the Netherlands (Graamans et al., 2018). Considering a vertical system (e.g., up to 10 tiers), this would allow to increase yield up to 25-folds the values obtained in greenhouses. Limited real environment applications are to date available, however. In indoor grown basil, average plant growth rate was reported to range 1–2 g plant^−1^ d^−1^ (Piovene et al., 2015), overall resembling similar values to those observed inside greenhouses (Saha et al., 2016). Growth rates in this study were consistent, ranging from 0.7 (RB ≤ 1) and 0.8 (CK_1_) to 1.2 (RB ≥ 2) g plant^−1^ d^−1^. However, due to the elevate planting density (100 plants m^−2^), when a vertical system of 5 or 10 tiers is adopted (Figure 1E), daily yield per surface of land occupied may increase up to, respectively, 567 and 1135 g m^−2^ day^−1^, when RB ≥ 2 is used. Although potential yields may even be increased when moving from an experimental unit to a commercial plant, these figures are already impressive, if compared with previous average growth rate of about 24–110 g m^−2^ day^−1^ recorded in traditional greenhouse production (Saha et al., 2016; Montesano et al., 2018). Moreover, such a difference may even increase due to the variability and reduction of the incident radiation as a consequence of seasonality in greenhouse grown crops.

#### Nutrient Use Efficiency

Similarly to the water use efficiency, NUE can be fostered by using hydroponics (Putra and Yuliando, 2015). Significant differences in nutrient uptake and accumulation among light treatments were observed (Table 2). In previous reports, the adoption of blue light in broccoli microgreen, resulted in greater accumulation of P, K, Ca, Mg, and Fe in leaf tissues as compared with plants grown under mixed red and blue LED (RB = 7), and this was associated with the increased transpirational fluxes and stomatal conductance under blue light (Kopsell and Sams, 2013). Altered fluxes of Ca and K in *Arabidopsis* were also associated with blue light (Babourina et al., 2002). A study on lettuce (Amoozgar et al., 2017) suggested that blue light increases accumulation of K and Ca, whereas a light source providing either a red or a mixture of red and blue (RB = 2) would result in increased level of Fe, P, Mg, and N accumulated in leaf tissues. Red light was also shown to increase N, P, K, and Mg uptake in lettuce (Pinho et al., 2017). On the other hand, no differences in N accumulation in lettuce leaves were found among monochromatic blue or red lights or mixtures of red and blue at varying ratio (e.g., RB = 0.5, 1, 3, and 13) (Clavijo-Herrera et al., 2018). Similarly, in this study, no differences in leaf N concentration was found in basil plants grown under LED regardless to the R:B ratio adopted (Table 2). Alternatively, when incident light at RB ≤ 1 resulted in the lowest growth performances, a concurrent and greater decrease in nutrient content (mg plant^−1^) was also observed. Accordingly, an increase in NUE in measure of +10% was observed as compared with CK_1_ and the other treatments (RB ≥ 2) (Figure 1F).

## Conclusion

Since the first LED applications in plant cultivation (Bula et al., 1991), the associated research has increased steadily, until it started to grow dramatically in recent years while vertical farms with artificial light are being implemented in North-America, Asia, and Europe (Kozai, 2015). In this new emerging sector, replicability of scientific evidences is often constrained by inconsistencies in the experimental setups, often resulting in simultaneous variations of multiple spectral parameters (e.g., including white, green, orange, and lime portions), lack of clear description of the spectral properties of the lamps used, or the adoption of limiting lighting conditions (e.g., low PPFDs). In order to build new and reliable knowledge in the sector, it is therefore crucial to implement linear and progressive research lines, allowing to identify optimal ratio among spectral components, their adaptability to the different crop phenological stages and the optimal light intensities, always paying attention to the sustainable use of resources, such as water, energy, land used, and minerals supplied. The present research can be seen as a first step toward this approach, identifying the optimal R:B ratio for sustainable indoor cultivation of sweet basil. A R:B ratio of 3 enabled to achieve highest yield, quality and resource use efficiency. In plants grown under RB_3_ a reduction in stomatal conductance combined with preserved plant growth resulted in increased water use efficiency. Concurrently, the higher yield associated with RB_3_ resulted in greater energy use efficiency, despite the reduction of PPNE observed in this treatment associated with working temperature of the lamps. On the other hand, the spectra with larger blue fractions (e.g., RB ≤ 1) resulted in lower yield and chlorophyll content, altogether with inefficiencies in water, energy and land surface use. Preliminary results on both photosynthetic quantum yield efficiency and volatile contents were also associated with changes in the light spectral composition, although further researches would be needed in order to confirm the observed trends. Starting from the promising results associated with RB_3_, future researches should target the effect of additional spectral regions, the identification of crop intra- and inter-specific variability in the response, as well as the definition of optimal light intensities. Crop management practices can also be addressed, e.g., adapting irrigation or fertilization plans to plant needs as they are affected by light quality.

## Author Contributions

GP designed and performed all experiments and drafted the manuscript. FO contributed to the experimental design and the drafting of the manuscript. SB and IB performed analyses of volatiles and nutrients and revised the manuscript. ACe and FS performed the analyses of antioxidants, contributed to the statistical analysis and revised the manuscript. LoM contributed to the experimental setup and performed measurements on energy use efficiency. ACr coordinated the manufacturing of the lamps used in the experiments. CS and LeM contributed to the experiments on PSII quantum efficiency and supported drafting the manuscript. SN, JF, and GG critically revised the manuscript.

## Conflict of Interest Statement

ACr was employed by company Flytech s.r.l. The remaining authors declare that the research was conducted in the absence of any commercial or financial relationships that could be construed as a potential conflict of interest.
