# Supplementary material for: Unraveling the Role of Red:Blue LED Lights on Resource Use Efficiency and Nutritional Properties of Indoor Grown Sweet Basil
Source: Front Plant Sci. 2019 Mar 13;10:305. doi: 10.3389/fpls.2019.00305 (PMC6424884; doi:10.3389/fpls.2019.00305)
Supplement: Supplementary file 1 [file Data_Sheet_1.docx]

Supplementary Material

**Unravelling the role of red:blue LED lights on resource use efficiency and nutritional properties of indoor grown sweet basil**

Giuseppina Pennisi^1,2,3^, Sonia Blasioli^1^, Antonio Cellini^1^, Lorenzo Maia^1^, Andrea Crepaldi^4^, Ilaria Braschi^1^, Francesco Spinelli^1^, Silvana Nicola^2^, Juan A. Fernandez^3^, Cecilia Stanghellini^5^, Leo F.M. Marcelis^6^, Francesco Orsini^1,6,*^, Giorgio Gianquinto^1^

*** Correspondence:** Corresponding Author: f.orsini@unibo.it

## Supplementary Figures and Tables


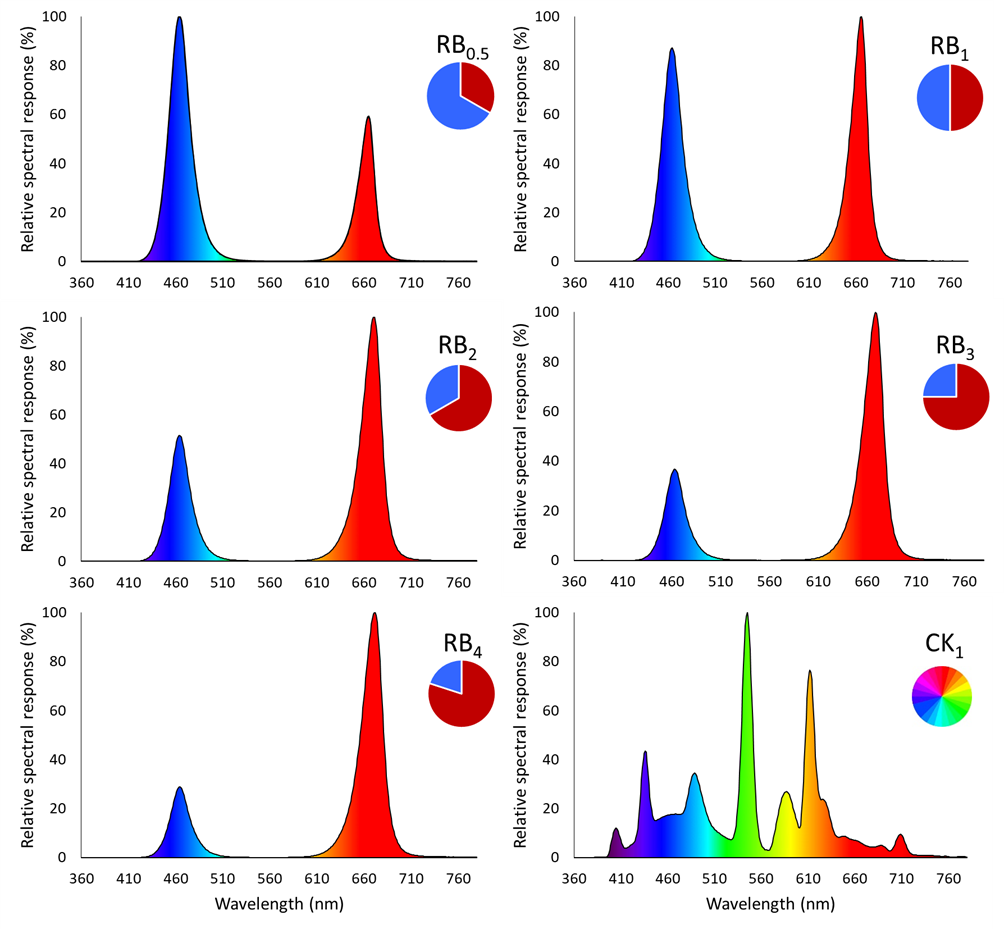


**Supplementary Figure 1.** Light spectra used in the experiments.

**Supplementary Table 1.** Experimental design and P values for the main and interactive effects of light and experiment based on two-ways Analysis of Variance (ANOVA). * significant differences at P≤0.05. In each experiment, all measurements were based on 9 replicate plants.

| Measure | Unit | exp. 1 | exp. 2 | exp. 3 | exp. 4 | P values (light) | P values  (exp.) | P values (light x exp.) |
| --- | --- | --- | --- | --- | --- | --- | --- | --- |
| Fresh weight | g plant^-1^ | X | X | X | X | <0.001* | <0.001* | 0.241 |
| Chlorophyll content | N tester units | X | X | X | X | <0.001* | 0.003* | 0.188 |
| EUE | g FW kW^-1^ | X | X | X | X | <0.001* | <0.001* | 0.120 |
| WUE | g FW g^-1^ H_2_O | X | X | X | X | <0.001* | 0.067 | 0.116 |
| SUE | g FW m^-2^ year^-1^ | X | X | X | X | <0.001* | <0.001* | 0.241 |
| NUE | g FW g^-1^ nutrients |  | X | X |  | <0.001* | 0.776 | 0.989 |
| Stomatal conductance | mmol H_2_O m^-2^ s^-1^ |  |  | X | X | <0.001* | <0.001* | 0.137 |
| Total flavonoid content | mg CE g^-1^ FW |  | X | X |  | <0.001* | <0.001* | 0.066 |
| Total polyphenols content | mg GA g^-1^ FW |  | X | X |  | 0.744 | 0.286 | 0.911 |
| FRAP | mmol Fe^2+^ kg^-1^ FW |  | X | X |  | 0.034* | 0.253 | 0.723 |
| N uptake | mg plant^-1^ |  | X | X |  | <0.001* | 0.108 | 0.416 |
| P uptake | mg plant^-1^ |  | X | X |  | <0.001* | 0.667 | 0.193 |
| K uptake | mg plant^-1^ |  | X | X |  | <0.001* | 0.235 | 0.337 |
| Ca uptake | mg plant^-1^ |  | X | X |  | 0.156 | 0.102 | 0.415 |
| Mg uptake | mg plant^-1^ |  | X | X |  | <0.001* | 0.584 | 0.727 |
| Fe uptake | mg plant^-1^ |  | X | X |  | <0.001* | <0.001* | 0.535 |
| N content in leaves | mg plant^-1^ |  | X | X |  | <0.001* | 0.378 | 0.971 |
| P content in leaves | mg plant^-1^ |  | X | X |  | <0.001* | 0.195 | 0.860 |
| K content in leaves | mg plant^-1^ |  | X | X |  | <0.001* | 0.252 | 0.914 |
| Ca content in leaves | mg plant^-1^ |  | X | X |  | <0.001* | 0.504 | 0.987 |
| Mg content in leaves | mg plant^-1^ |  | X | X |  | 0.002* | 0.601 | 0.993 |
| Fe content in leaves | mg plant^-1^ |  | X | X |  | <0.001* | 0.016 | 0.188 |

**Supplementary Table 2.** Volatile compounds found in basil plants grown under LED lights with different R:B ratio in the spectrum (RB_0.5_, RB_1_, RB_2_, RB_3_ and RB_4_) or under fluorescent lights (CK_1_) expressed as % of the total content (mean ± SE of three replicate plants, exp. 3). RT=Retention time.

| Compound | RT  (min) | CK_1_  (%) | RB_0.5_  (%) | RB_1_  (%) | RB_2_  (%) | RB_3_  (%) | RB_4_ (%) |
| --- | --- | --- | --- | --- | --- | --- | --- |
| α-pinene | 4.026 | 0.5±0.3 | 1.4±0.8 | 0.5±0.2 | 0.3±0.0 | 0.9±0.3 | 0.6±0.2 |
| α-phellandrene | 4.158 | 0.3±0.1 | 2.0±0.9 | 0.2±0.0 | 0.2±0.1 | 0.5±0.2 | 0.3±0.1 |
| toluene | 4.347 | 0.1±0.0 | 0.7±0.6 | 0.1±0.0 | 0.1±0.0 | 0.1±0.0 | 0.1±0.1 |
| camphene | 4.892 | 0.0±0.0 | 0.2±0.1 | 0.0±0.0 | 0.0±0.0 | 0.1±0.1 | 0.0±0.0 |
| β-pinene | 6.360 | 0.8±0.4 | 3.2±1.2 | 0.4±0.2 | 0.5±0.1 | 0.9±0.3 | 0.8±0.3 |
| β-phellandrene | 6.993 | 2.2±0.8 | 3.2±2.0 | 0.8±0.4 | 1.3±0.2 | 2.1±1.1 | 2.0±0.2 |
| 3-carene | 8.261 | 0.0±0.0 | 0.5±0.2 | 0.0±0.0 | 0.0±0.0 | 0.3±0.2 | 0.0±0.0 |
| myrcene | 9.573 | 3.7±0.3 | 3.9±2.4 | 3.0±0.5 | 2.9±0.3 | 4.7±1.7 | 4.0±0.4 |
| terpinolene | 10.055 | 1.7±0.4 | 3.4±0.4 | 1.1±0.2 | 1.1±0.2 | 2.3±0.8 | 1.5±0.3 |
| d-limonene | 11.196 | 1.8±0.4 | 3.4±0.4 | 1.2±0.2 | 1.2±0.1 | 2.0±0.5 | 1.9±0.3 |
| eucalyptol | 11.592 | 1.0±0.4 | 2.2±0.8 | 2.4±0.1 | 0.7±0.1 | 2.3±1.0 | 1.7±0.6 |
| β-*trans*-ocimene | 13.787 | 0.3±0.1 | 0.2±0.2 | 0.1±0.1 | 0.3±0.0 | 0.6±0.1 | 0.4±0.0 |
| γ-terpinene | 13.900 | 0.2±0.1 | 0.6±0.1 | 0.2±0.0 | 0.2±0.0 | 0.3±0.1 | 0.2±0.0 |
| β-*cis*-ocimene | 14.589 | 7.1±3.9 | 12.9±1.9 | 8.5±1.8 | 9.1±1.7 | 12.2±2.4 | 11.9±1.1 |
| P-cimene | 15.057 | 0.0±0.0 | 0.2±0.0 | 0.1±0.0 | 0.1±0.0 | 0.0±0.0 | 0.1±0.0 |
| geranyl nitrile | 17.322 | 1.9±1.1 | 0.0±0.0 | 0.0±0.0 | 0.0±0.0 | 0.3±0.3 | 0.6±0.3 |
| 3-hexenyl acetate | 18.971 | 0.1±0.1 | 0.1±0.1 | 0.0±0.0 | 0.0±0.0 | 0.1±0.1 | 0.0±0.0 |
| octan-3-ol | 20.983 | 0.2±0.0 | 0.1±0.1 | 0.1±0.1 | 0.1±0.0 | 0.1±0.1 | 0.1±0.0 |
| 1-octen-3-ol | 23.006 | 0.3±0.1 | 0.5±0.1 | 0.4±0.2 | 0.2±0.2 | 0.0±0.0 | 0.2±0.2 |
| β-terpineol | 23.291 | 0.1±0.0 | 0.1±0.0 | 0.2±0.1 | 0.1±0.0 | 0.0±0.0 | 0.0±0.0 |
| copaene | 24.085 | 0.3±0.1 | 0.6±0.2 | 0.5±0.1 | 0.4±0.1 | 0.6±0.0 | 0.6±0.2 |
| α-cubebene | 25.709 | 0.6±0.1 | 0.5±0.1 | 0.6±0.1 | 0.4±0.0 | 0.2±0.2 | 0.7±0.2 |
| zingiberene | 25.851 | 0.1±0.0 | 0.2±0.0 | 0.2±0.0 | 0.2±0.0 | 0.1±0.0 | 0.1±0.0 |
| linalool | 26.322 | 44.9±1.8 | 12.8±3.1 | 37.1±3.4 | 42.7±6.0 | 35.9±2.6 | 38.6±5.1 |
| linalyl formate | 26.640 | 0.0±0.0 | 0.1±0.0 | 0.0±0.0 | 0.1±0.1 | 0.1±0.0 | 0.1±0.1 |
| bornyl acetate | 26.950 | 0.0±0.0 | 0.1±0.0 | 0.1±0.0 | 0.1±0.0 | 0.0±0.0 | 0.0±0.0 |
| allo-aromadendrene | 29.116 | 0.1±0.0 | 0.0±0.0 | 2.4±0.4 | 2.5±0.4 | 0.1±0.0 | 0.2±0.1 |
| humulene | 29.576 | 0.9±0.1 | 1.0±0.3 | 1.0±0.2 | 0.7±0.1 | 1.0±0.2 | 1.4±0.8 |
| terpin hydrate | 29.791 | 0.1±0.1 | 0.2±0.0 | 0.1±0.1 | 0.1±0.1 | 0.1±0.1 | 0.1±0.1 |
| β-farnesene | 30.000 | 1.7±0.3 | 3.8±0.9 | 3.0±0.5 | 2.4±1.0 | 5.1±1.3 | 2.5±1.0 |
| α-terpineol | 30.533 | 0.9±0.2 | 0.9±0.1 | 1.0±0.3 | 0.6±0.1 | 0.6±0.1 | 1.0±0.1 |
| β-cubebene | 30.770 | 2.4±0.3 | 2.5±0.6 | 3.3±0.9 | 2.4±0.1 | 2.6±0.3 | 3.5±1.3 |
| α-bulnesene | 31.115 | 0.9±0.2 | 0.6±0.2 | 0.7±0.2 | 0.6±0.0 | 0.7±0.1 | 1.1±0.5 |
| γ-elemene | 31.473 | 0.1±0.1 | 0.3±0.1 | 0.7±0.2 | 0.5±0.1 | 0.0±0.0 | 0.6±0.5 |
| β-bisabolene | 31.574 | 0.1±0.0 | 0.1±0.0 | 0.1±0.0 | 0.1±0.0 | 0.1±0.0 | 0.1±0.0 |
| α-bergamotene | 31.959 | 17.9±1.1 | 28.6±5.9 | 25.1±1.6 | 23.5±2.5 | 17.0±3.4 | 16.9±1.6 |
| γ-muurolene | 32.272 | 3.2±0.6 | 3.0±0.8 | 3.6±0.9 | 2.8±0.2 | 3.2±0.4 | 3.9±1.1 |
| α-cadinene | 33.187 | 0.2±0.0 | 0.1±0.0 | 0.2±0.1 | 0.1±0.0 | 0.2±0.0 | 0.2±0.1 |
| calamenene | 34.183 | 0.2±0.1 | 0.2±0.0 | 0.3±0.1 | 0.1±0.1 | 0.2±0.0 | 0.3±0.1 |
| methyl eugenol | 39.045 | 0.0±0.0 | 3.8±2.6 | 0.0±0.0 | 0.0±0.0 | 0.4±0.1 | 0.0±0.0 |
| isoeugenol | 42.402 | 2.1±1.0 | 1.4±0.7 | 0.4±0.1 | 0.6±0.2 | 1.2±0.3 | 0.6±0.2 |
| β-gurjunene | 42.660 | 1.0±0.2 | 0.6±0.3 | 0.5±0.1 | 0.7±0.2 | 0.9±0.2 | 1.0±0.3 |
